# Guidelines for Treatment of Umbilical and Epigastric Hernias From the European and Americas Hernia Societies–A Web-Based Survey on Surgeons’ Opinion

**DOI:** 10.3389/jaws.2022.10260

**Published:** 2022-03-21

**Authors:** N. A. Henriksen, T. Nazari, M. P. Simons, W. Hope, A. Montgomery

**Affiliations:** ^1^ Department of Gastrointestinal and Liver Diseases, Herlev Hospital, Herlev, Denmark; ^2^ Faculty of Medicine, University of Copenhagen, Copenhagen, Denmark; ^3^ Department of Surgery, Erasmus Medical Center, Erasmus University Rotterdam, Rotterdam, Netherlands; ^4^ Onze Lieve Vrouwe Gasthuis (OLVG), Amsterdam, Netherlands; ^5^ New Hanover Regional Medical Center, Wilmington, DE, United States; ^6^ Faculty of Medicine, Department of Surgery, Skane University Hospital, Malmö, Sweden

**Keywords:** survey, umbilical, epigastric, voting, level of consensus, SurveyMonkey

## Abstract

**Background and aims:** The European and Americas Hernia Society’s (EHS and AHS) Guidelines on the treatment of primary midline ventral hernias were launched to guide surgeons. As a part of a dissemination plan of the guideline, this study aimed to evaluate the level of consensus between recommendations and the current surgical practices of EHS and AHS members before implementation.

**Material and methods:** A questionnaire was constructed including questions on the current practice of the members and nine selected key recommendations from the guidelines. An on-stage consensus voting was performed at the EHS Congress in Hamburg 2019 followed by a SurveyMonkey sent to all EHS and AHS members. Consensus with a recommendation was defined as an agreement of ≥70%.

**Results:** A total of 178 votes were collected in Hamburg*.* A further 499/1,754 (28.4%) of EHS and 150/1,100 (13.6%) of AHS members participated in the SurveyMonkey. A consensus was reached for 7/9 (78%) of the recommendations. The two recommendations that did not reach consensus were on indication and the technique used for laparoscopic repair. In current practice, more AHS participants used a preformed patch; 50.7% (76/150) compared with EHS participants 32.1% (160/499), *p* < 0.001.

**Conclusion:** A consensus was achieved for most recommendations given by the new guideline for the treatment of umbilical and epigastric hernias. Recommendations that did not reach consensus were on indication and technique for laparoscopic repair, which may reflect the lack of evidence on these topics.

## Introduction

The European Hernia Society (EHS) has published guidelines for the treatment of different types of abdominal wall hernias in recent decades. These guidelines enable hernia surgeons to use evidence-based indications for surgery as well as operative techniques (1–3). The last published guideline from the EHS was in collaboration with the Americas Hernia Society (AHS) on the treatment of primary ventral hernias (4). New guidelines are commonly launched at the annual EHS or combined EHS/AHS congresses and are being discussed with hernia surgeons from around the world.

It is difficult to implement new routines in surgical practice, even though there is evidence available indicating that practices should be changed (4). It is unknown whether this is due to discomfort with behavioral changes in general, disagreement with the recommendations of the published guidelines, the belief that current practice is better, or a lack of awareness of the published guidelines.

As a part of a dissemination plan of the guideline, the current study aimed to assess the opinion of EHS and AHS members, representing the general hernia surgeons, on evidence-based recommendations for treatment strategies of primary ventral hernias (4). An on-stage consensus voting was undertaken at the EHS congress in Hamburg in 2019 followed by a SurveyMonkey sent to all EHS and AHS members in the Summer of 2020 to measure the level of consensus between the newly published evidence-based recommendations of the societies and current surgical practice of EHS and AHS members in connection with the implementation of these primary midline ventral hernia guidelines.

## Material and Methods

The study was based on the recently developed EHS and AHS joint guidelines on primary midline ventral hernias. It consists of 18 key questions (KQ) including both indications for surgery and different surgical strategies/techniques (5).

The questionnaire was constructed by five participants involved with the guidelines working group. The questionnaire included a few initial questions on the current practice of the EHS and AHS members, considering the use of mesh for hernias with a defect <1 cm, and in case using mesh, the type used. Nine potentially controversial (out of the 18) KQs from the guideline were selected by the group to be included in the current consensus voting (Supplementary Appendix S1). These nine specific KQ were chosen as they had either been the subject of discussions during the creation of the guidelines or because we anticipated that the community might find them difficult to accept. The KQs that were not included were related to diagnostics, preoperative optimization, antibiotics, suture technique, emergency repair, mesh fixation at open repair, and type of anesthesia. The nine included KQs are listed in Table 1 (we used the “original KQ number” to avoid the publications becoming mixed up) (5).

**TABLE 1:** Key questions (KQ) included in consensus voting. The numbers used are derived from the published guidelines.

KQ 1: What is the definition of an umbilical hernia and an epigastric hernia?
KQ 3: Is a watchful waiting strategy safe in patients with asymptomatic umbilical or epigastric hernias?
KQ 6: Is there a place for sutured repair in elective umbilical or epigastric hernia repair?
KQ 9: Which is the preferred type of mesh and the preferred layer for mesh placement when doing an open umbilical or epigastric hernia repair?
KQ 10: Which is the preferred mesh overlap for open umbilical or epigastric hernia repair?
KQ 12: Should the defect be closed for open umbilical and epigastric hernia repairs when using a mesh?
KQ 14: What are the indications for laparoscopic umbilical and epigastric hernia repair?
KQ 15: What is the preferred laparo-endoscopic repair method for umbilical or epigastric hernias?
KQ 16: Which is the preferred repair method of umbilical and epigastric hernias based on hernia and patient characteristics?

Four options were available for the consensus voting:

1. Agree with recommendation and strength of recommendation
2. Agree with recommendation only
3. Disagree with recommendation
4. Don’t know.

The consensus was defined as a positive answer to either option 1 (Agree with recommendation and strength of recommendation) or 2 (Agree with recommendation only).

### Consensus Voting at EHS Congress

At the EHS Congress in Hamburg on 12 September 2019, the Guidelines for Primary Midline Ventral Hernias were presented on-stage for the first time by the participants of the guideline group. The evidence for both the statements and recommendations for all 18 KQs were presented. The audience voted on their level of agreement with the nine selected KQs recommendations using an online app.

### SurveyMonkey–Online Voting

A SurveyMonkey was created, including the initial questions described above together with the statements and the recommendations of the nine selected KQs, and sent by mail to all members of the EHS and AHS available on the mailing lists (Supplementary Appendix S1). The published guideline paper was attached to the email. The first email was sent on 22 May 2020. A reminder was then emailed on 15 June and 11 August 2020, respectively.

### Statistics

The degree of consensus (DoC) was reported separately for the on-stage voting at the EHS congress in Hamburg 2019, and for the EHS and AHS society members for online voting. “Overall consensus” was defined as an average of the result of these three votes showing an agreement of >70% (6). The Level of Evidence (LoE) was reported according to the original guideline’s assessment of evidence, which was either very low or high (5, 6). The results from EHS and AHS participants were compared with the Chi^2^ test in Excel and a *p* < 0.05 was considered statistically significant.

## Results

At the EHS congress in Hamburg, a total of 178 participated in the live consensus voting. The EHS and AHS members mailing lists included 1,754 and 1,100 members, respectively. A total of 28.4% (499/1,754) and 13.6% (150/1,100) of the contacted members of the EHS and AHS participated in the SurveyMonkey consensus voting, respectively. Overall, the response rate of the SurveyMonkey consensus voting was 15.7% (649/2,854).

**FIGURE 1:**
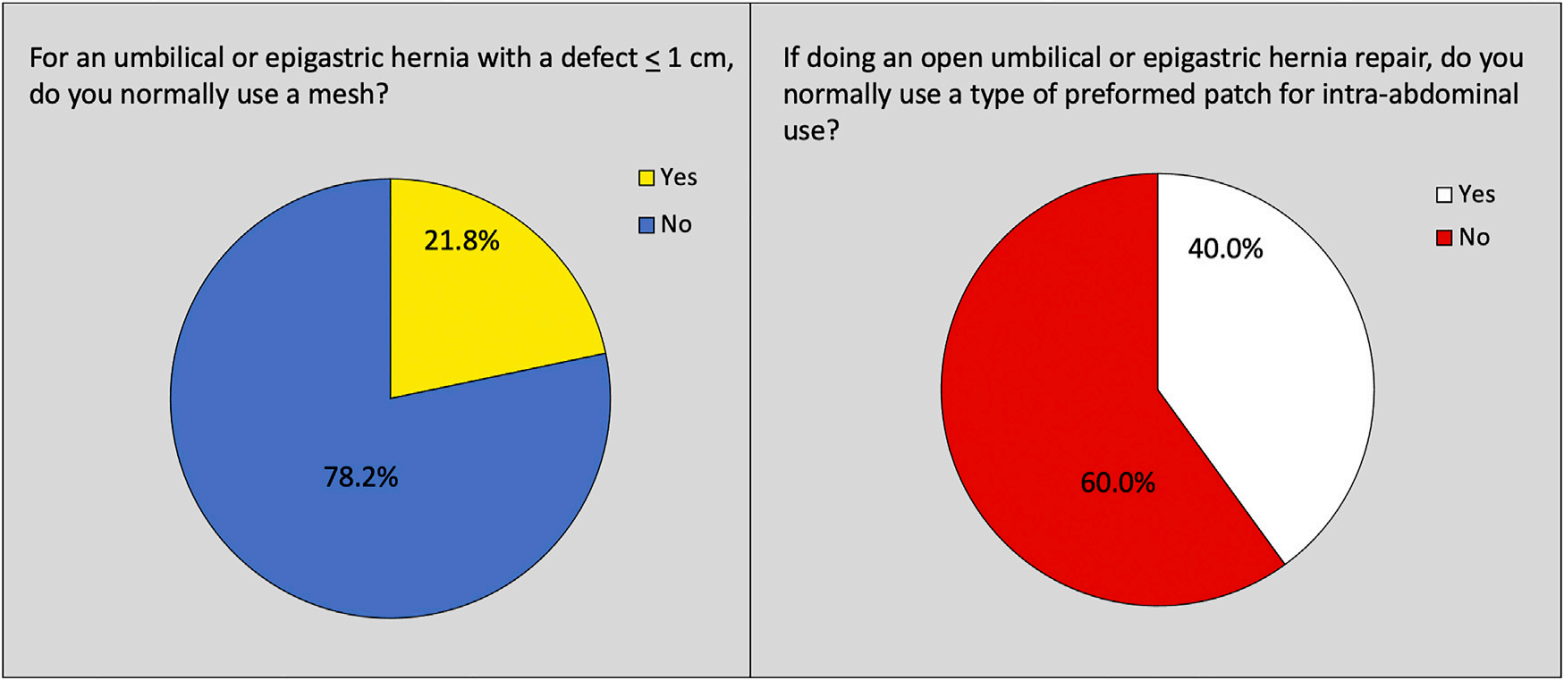
Questions reflecting current practice on the use of mesh and mesh type for umbilical and epigastric hernia repair.

For the first initial question on current practice, 21.8% (180/827) of the participants “normally used a mesh” for umbilical and epigastric hernias with defects ≤1 cm (Figure 1). There was no difference on the use of mesh for defects ≤1 cm between EHS (21.0%, 105/499) and AHS (23.3% 35/150) participants, *p* = 0.421. A total of 40.0% (331/827) normally used a preformed patch when using a mesh for open repair of umbilical and epigastric hernias (Figure 1). More AHS participants used a preformed patch 50.7% (76/150) compared with EHS participants 32.1% (160/499), *p* < 0.001.

**FIGURE 2:**
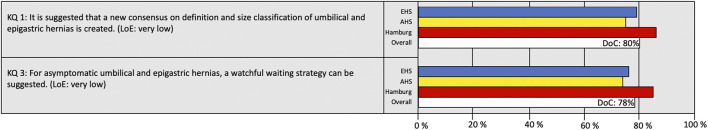
Degree of consensus (DoC) for recommendations on definition and watchful waiting. LoE, Level of Evidence. Definition of consensus: >70% agreement (6).

**FIGURE 3:**
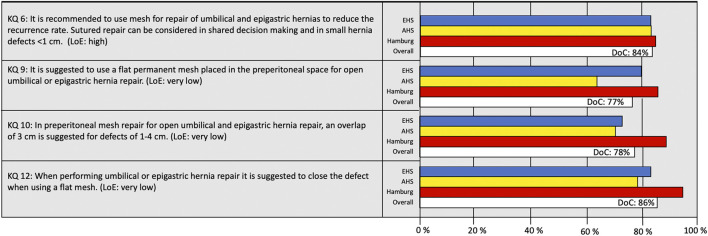
Degree of consensus (DoC) for recommendations on open repairs. LoE, Level of Evidence. Definition of consensus: >70% agreement (6).

The highest DoC was reached at the live vote at the EHS congress in Hamburg. A consensus was reached for 78% (7/9) of the recommendations. For the initial recommendations on the definition and watchful waiting, a consensus was reached with a DoC of 86% and 78%, respectively (Figure 2). Considering surgical techniques for open umbilical and epigastric hernia repairs, the consensus was reached for all recommendations (Figure 3).

**FIGURE 4:**
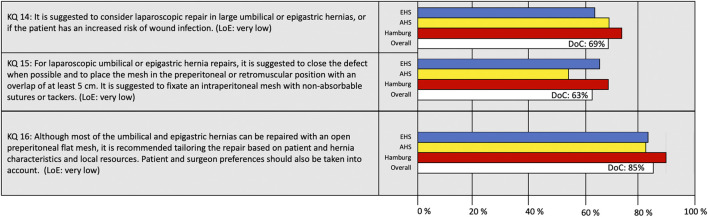
Degree of consensus (DoC) for recommendations on laparoscopic repairs and tailored approach. LoE, Level of Evidence. Definition of consensus: >70% agreement (6).

For eight of the nine included recommendations, the LoE was very low (Figures 2–4). For KQ 9, the recommendation on placing a flat mesh in the preperitoneal space for open umbilical and epigastric hernia repair, the DoC was significantly lower for AHS (64%; 96/150) compared with EHS participants (80%;399/499), *p* < 0.001.

Considering “when to use a laparoscopic approach” and “which technique to use,” a consensus was not reached for either recommendation (Figure 4). The recommendation of KQ 15 on the preferred laparo-endoscopic technique addressed both defect closure, mesh overlap, mesh layer, and fixation technique, leading to an overall DoC of 63%. The last KQ on tailored approach reached a DoC of 85%.

## Discussion

This consensus survey, including members of two major hernia societies, was performed to evaluate the surgeons’ current practice and opinion on the recently published EHS and AHS guidelines on the treatment of primary midline ventral hernias.

The study is strengthened by the fact that it includes an online survey, distributed to many participants giving surgeons with a special interest in hernias the possibility to both read and critically review the guideline. However, only 15.7% participated, despite two reminders. This is a major limitation of the study. Nevertheless, this response rate is comparable to other available SurveyMonkey studies sent to surgeons with a reported response rate of 8.8–26% (7–9). It is unknown whether a higher response rate would have altered the DoC.

The proportion of European surgeons participating in the survey was significantly higher than the proportion of participants from the American continent. One explanation for this might be that the results of the guidelines were presented on stage at the EHS congress in Hamburg 2019, thereby exposing more European hernia surgeons to these guidelines. Also, the number of EHS (57%) members exceeds those of AHS (43%) in total.

A consensus was defined as an agreement with the recommendation ≥70%, which was reached for 78% (7/9) of the recommendations, which must be regarded as acceptable considering the most controversial questions were chosen to be included in this SurveyMonkey. The highest DoC was reached at the live vote at the EHS congress in Hamburg, which may be explained by the fact that the reasons for and the methodology behind a given recommendation were explained in more detail before voting. Even though the guidelines paper was included in the email with the SurveyMonkey, it is possible that the respondents of the SurveyMonkey did not read the paper in detail or at all.

A consensus was reached on the definition of primary ventral hernias and the recommended surgical technique for open repairs as well as the recommendation to use a tailored approach (Figures 2, 3). The two recommendations not reaching consensus were indications for laparoscopic surgery and techniques used for laparoscopic repair (KQ 14 and 15).

Indication for surgery overall is always difficult to agree on. It could alter between both individual surgeons’ case mix and surgeons and there being different incentives for indications to recommend operation or not. Indications for the laparoscopic technique are dependent on the surgeon’s expertise and the availability of equipment for laparoscopic surgery. Considering the recommendation on laparoscopic technique, the DoC was only 63% (KQ 15, Figure 4). This lack of consensus may be explained by the fact that this recommendation included several entities such as defect closure, mesh overlap, layer for mesh placement, and mesh fixation, making it impossible to agree on only a part of the recommendation. Therefore, composite questions cannot be recommended to be used in a consensus survey.

The available evidence on laparoscopic repair for umbilical and epigastric hernias is limited and the majority of available studies included both primary ventral and incisional hernias (10, 11). It is difficult to extrapolate data from incisional hernias directly to primary ventral hernias. For a primary umbilical hernia with a defect of 2 cm, it may seem “exaggerated” to use a laparoscopic approach and insert a large mesh. However, it does seem clear that laparoscopic repair decreases the risk of wound complication compared with open repair (12). Based on this low LoE, the guideline group recommended using laparoscopic repair only for larger defects and in patients at high risk of wound complications. A consensus on this was almost reached, with a LoC of 69%. This lack of consensus may be explained by the emerging use of newer laparoscopic techniques (eTEP, MILOS, eMILOS, and robot-assisted repairs), which may have advantages compared with open repairs and standard laparoscopic intraperitoneal onlay mesh (IPOM), though data are scarce (13–15).

There was a significant difference between EHS and AHS participants concerning the use of preformed patches for open umbilical and epigastric hernias, with a dominance of preformed patches used by AHS participants. Half of the participating surgeons from AHS used a preformed patch for the open repair of umbilical and/or epigastric hernias. This led to a lack of consensus among surgeons from the AHS on the recommendation to use a flat mesh for open repairs. This difference may be explained by cultural differences as well as differences in healthcare and reimbursement systems. The preformed patches are easier to use and shorten operating time, which may also be a key factor (16, 17). Nevertheless, both early and late outcomes have been shown to be poorer with the use of a patch compared to a flat mesh. Case series describe serious long-term complications with patches, which formed the basis for the guideline recommendations (16–19). It will of course be interesting to see whether this recommendation will change the practice of EHS and AHS surgeons over time.

A consensus survey is important as it engages surgeons of the hernia societies to express their opinion. It will raise curiosity to explore new fields and question oneself regarding the best choices for patients. Furthermore, it is essential to assess recommendations that are controversial to the community and promote further research. Lastly, raising awareness of a recently published guideline is a part of disseminating guidelines to the surgical community.

In conclusion, an overall high rate of consensus was reached among EHS and AHS members on the guidelines for the treatment of umbilical and epigastric hernias. The use of a preformed patch was more commonly used by surgeons from America compared to Europe. The recommendations that did not reach consensus were on indication and technique for laparoscopic repair, which may reflect the lack of evidence on this topic. Dissemination and implementation of guidelines are important to promote evidence-based practices and to clarify areas where research is lacking.

## Data Availability

The raw data supporting the conclusion of this article will be made available by the authors, without undue reservation.
