# Supplementary material for: Guidelines for Treatment of Umbilical and Epigastric Hernias From the European and Americas Hernia Societies–A Web-Based Survey on Surgeons’ Opinion
Source: J Abdom Wall Surg. 2022 Mar 21;1:10260. doi: 10.3389/jaws.2022.10260 (PMC10831711; doi:10.3389/jaws.2022.10260)
Supplement: Supplementary file 1 [file DataSheet1.PDF]

Brand New Ventral Hernia Guidelines - Evidence and Consensus voting

Questions presented to the audience at the symposium in EHS Hamburg

* Did you attend the Ventral Hernia Guidelines - Evidence and Consensus voting session during the European Hernia Society Congress in Hamburg on the 12th of September?

◯ Yes

◯ No

* For an umbilical or epigastric hernia with a defect ≤ 1 cm, do you normally use a mesh?

◯ Yes

◯ No

* If doing an open mesh repair of an umbilical or epigastric hernia, do you normally use a type of preformed ventral patch for intra-abdominal use?

◯ Yes

◯ No

* If doing an open mesh repair of an umbilical or epigastric hernia, do you normally use a flat preperitoneal mesh?

◯ Yes

◯ No

Brand New Ventral Hernia Guidelines - Evidence and Consensus voting

Questions presented to the audience at the symposium in EHS Hamburg

What is the definition of an umbilical hernia and an epigastric hernia?

Statement: According to the classification for ventral hernias from the EHS, an umbilical hernia is defined as a hernia with the defect located in the midline at the umbilical level within 3 cm of the umbilicus. An epigastric hernia is defined as a hernia with the defect located in the midline from the 3 cm above the umbilicus up to the xiphoid process. Umbilical and epigastric hernias may be divided into small (0-2 cm), medium (2-4 cm) and large (> 4 cm) based on the diameter of the defect.

* **Recommendation:** It is recommended to use the EHS classification system when defining umbilical and epigastric hernias.

Quality of evidence: ☒☐☐☐

Strength of recommendation: strong (upgraded)

- ◯ Agree with recommendation and strength of the recommendation
- ◯ Agree with recommendation only
- ◯ Disagree with recommendation
- ◯ Don’t know

Brand New Ventral Hernia Guidelines - Evidence and Consensus voting

Questions presented to the audience at the symposium in EHS Hamburg

Is a watchful waiting strategy safe in patients with asymptomatic umbilical or epigastric hernias?

Statement: There is limited data on watchful waiting for patients with umbilical and epigastric hernias, but a watchful waiting strategy seems safe.

* **Recommendation:** For asymptomatic umbilical and epigastric hernias, a watchful waiting strategy can be suggested.

Quality of evidence: ☒☐☐☐

Strength of recommendation: weak

- ◯ Agree with recommendation and strength of the recommendation
- ◯ Agree with recommendation only
- ◯ Disagree with recommendation
- ◯ Don’t know

Brand New Ventral Hernia Guidelines - Evidence and Consensus voting

Questions presented to the audience at the symposium in EHS Hamburg

Is there a place for sutured repair in elective umbilical or epigastric hernia repair?

Statement: The use of mesh for open umbilical or epigastric hernia repair reduces the rate of recurrence without increasing the rate of surgical site infection. The quality of evidence is limited for hernias with defect sizes of 0-1 cm.

* **Recommendation:** It is recommended to use mesh for repair of umbilical and epigastric hernias to reduce the recurrence rate. Sutured repair can be considered in shared decision making and in small hernia defects <1 cm.

Quality of evidence: ☒☒☒☒

Strength of recommendation: strong

- ◯ Agree with recommendation and strength of the recommendation
- ◯ Agree with recommendation only
- ◯ Disagree with recommendation
- ◯ Don't know

Brand New Ventral Hernia Guidelines - Evidence and Consensus voting

Questions presented to the audience at the symposium in EHS Hamburg

Which is the preferred type of mesh and the preferred layer for mesh placement when doing an open primary ventral hernia repair?

Statement: The use of intraperitoneal patches may shorten surgical time but may be associated with more complications compared with a flat mesh placed in the preperitoneal layer. Patches or pre-shaped prosthetics with anti-adhesive barriers are more expensive than a synthetic flat mesh. Acceptable evidence finds that placement of the mesh in the retromuscular or preperitoneal position is associated with a lower rate of surgical site infection and recurrence. Placement of the mesh in the preperitoneal space seems safe and feasible.

* **Recommendation:** It is suggested to use a flat permanent mesh placed in the preperitoneal space for open umbilical or epigastric hernia repair.

Quality of evidence: ☒☐☐☐

Strength of recommendation: weak

○ Agree with recommendation and strength of the recommendation

○ Agree with recommendation only

○ Disagree with recommendation

○ Don't know

EUROPEAN HERNIA SOCIETY

AMERICAS HERNIA SOCIETY
North America ★ Central America ★ South America

Brand New Ventral Hernia Guidelines - Evidence and Consensus voting

Questions presented to the audience at the symposium in EHS Hamburg

Which is the preferred mesh overlap for open primary ventral hernia repair?

Statement: There is not enough evidence to recommend a specific mesh overlap that may reduce recurrences after umbilical and epigastric hernia repair. A preperitoneal mesh with an overlap of 3 cm has been associated with low recurrence rates in umbilical hernias. There is no evidence to make recommendations regarding the overlap in onlay or
intraperitoneal mesh placement.

* **Recommendation:** In preperitoneal mesh repair for open umbilical and epigastric hernia repair, an overlap of 3 cm is suggested.

Quality of evidence: ☒☐☐☐

Strength of recommendation: weak

- ◯ Agree with recommendation and strength of the recommendation
- ◯ Agree with recommendation only
- ◯ Disagree with recommendation
- ◯ Don’t know

Brand New Ventral Hernia Guidelines - Evidence and Consensus voting

Questions presented to the audience at the symposium in EHS Hamburg

Should the defect be closed for open umbilical and epigastric hernia repairs?

Statement: There is not enough evidence to recommend whether the defect should be left open or closed in open primary ventral hernia repair. Studies using a flat mesh have reported closing of the defect.

* **Recommendation:** When performing umbilical or epigastric hernia repair it is suggested to close the defect when using a flat mesh.

Quality of evidence: ☒☐☐☐

Strength of recommendation: weak

- ◯ Agree with recommendation and strength of the recommendation
- ◯ Agree with recommendation only
- ◯ Disagree with recommendation
- ◯ Don’t know

EUROPEAN HERNIA SOCIETY

AMERICAS HERNIA SOCIETY
North America ⋆ Central America ⋆ South America

Brand New Ventral Hernia Guidelines - Evidence and Consensus voting

Questions presented to the audience at the symposium in EHS Hamburg

What are the indications for laparoscopic repair?

Statement: Laparoscopic repair decreases the risk of wound complications. Laparoscopic repair may be beneficial in large umbilical or epigastric hernias. In small or medium-sized hernias, laparoscopic repair may be advantageous in patients at high risk of wound infection.

* **Recommendation:** It is suggested to consider laparoscopic repair in large umbilical or epigastric hernias, or if the patient has an increased risk of wound infection.

Quality of evidence: ☒☐☐☐

Strength of recommendation: weak

- ◯ Agree with recommendation and strength of the recommendation
- ◯ Agree with recommendation only
- ◯ Disagree with recommendation
- ◯ Don’t know

EUROPEAN HERNIA SOCIETY

AHS AMERICAS HERNIA SOCIETY
North America • Central America • South America

Brand New Ventral Hernia Guidelines - Evidence and Consensus voting

Questions presented to the audience at the symposium in EHS Hamburg

Which is the preferred laparoscopic repair method?

Statement: Novel laparoscopic techniques with extraperitoneal mesh placement seem promising with theoretical advantages to traditional IPOM technique, but there is currently insufficient data to suggest the superiority of one technique over another. As an intraperitoneal mesh may cause adhesions, an extraperitoneal mesh placement is suggested when possible. Closure of the defect seems to decrease seroma formation, bulging and recurrence. A mesh overlap of at least 5 cm seems to decrease recurrence rates. For IPOM repairs, fixation of the mesh with a non-absorbable device, either sutures or tackers decrease the recurrence rate.

* **Recommendation:** When doing a laparo-endoscopic umbilical or epigastric hernia repair, it is suggested to close the defect when possible and to place the mesh extraperitoneally with an overlap of at least 5 cm. It is suggested to fixate an intraperitoneal mesh with non-absorbable sutures or tackers.

Quality of evidence: ☒☐☐☐

Strength of recommendation: weak

- ○ Agree with recommendation and strength of the recommendation
- ○ Agree with recommendation only
- ○ Disagree with recommendation
- ○ Don’t know

EUROPEAN HERNIA SOCIETY

AMERICAS HERNIA SOCIETY
North America • Central America • South America

Brand New Ventral Hernia Guidelines - Evidence and Consensus voting

Questions presented to the audience at the symposium in EHS Hamburg

Which is the preferred repair method of umbilical and epigastric hernias based on defect size and patient characteristics?

Statement: Most umbilical and epigastric hernias have defects less than 2 cm and can be repaired with an open mesh repair. For larger defects or in patients with certain risk factors, laparoscopic repair may be used.

* **Recommendation:** Although most of the umbilical and epigastric hernias can be repaired with an open preperitoneal flat mesh, it is recommended tailoring the repair based on patient and hernia characteristics and local resources. Patient and surgeon preferences should also be taken into account.

Quality of evidence: ☒☐☐☐

Strength of recommendation: strong (upgraded)

- ○ Agree with recommendation and strength of the recommendation
- ○ Agree with recommendation only
- ○ Disagree with recommendation
- ○ Don’t know

Brand New Ventral Hernia Guidelines - Evidence and Consensus voting

Questions presented to the audience at the symposium in EHS Hamburg

* After reading the recommendations of the guideline, do you consider changing your practice?

- ◯ Yes
- ◯ No
